# How Large Should Whales Be?

**DOI:** 10.1371/journal.pone.0053967

**Published:** 2013-01-10

**Authors:** Aaron Clauset

**Affiliations:** 1 Department of Computer Science, University of Colorado, Boulder, Colorado, United States of America; 2 BioFrontiers Institute, University of Colorado, Boulder, Colorado, United States of America; 3 Santa Fe Institute, Santa Fe, New Mexico, United States of America; University of Namur, Belgium

## Abstract

The evolution and distribution of species body sizes for terrestrial mammals is well-explained by a macroevolutionary tradeoff between short-term selective advantages and long-term extinction risks from increased species body size, unfolding above the 2 *g* minimum size induced by thermoregulation in air. Here, we consider whether this same tradeoff, formalized as a constrained convection-reaction-diffusion system, can also explain the sizes of fully aquatic mammals, which have not previously been considered. By replacing the terrestrial minimum with a pelagic one, at roughly 7000 *g*, the terrestrial mammal tradeoff model accurately predicts, with no tunable parameters, the observed body masses of all extant cetacean species, including the 175,000,000 *g* Blue Whale. This strong agreement between theory and data suggests that a universal macroevolutionary tradeoff governs body size evolution for all mammals, regardless of their habitat. The dramatic sizes of cetaceans can thus be attributed mainly to the increased convective heat loss is water, which shifts the species size distribution upward and pushes its right tail into ranges inaccessible to terrestrial mammals. Under this macroevolutionary tradeoff, the largest expected species occurs where the rate at which smaller-bodied species move up into large-bodied niches approximately equals the rate at which extinction removes them.

## Introduction

Cetaceans include the largest animals ever to live, including the Blue Whale (*Balaenoptera musculus*), which is nearly 30 times larger than an African elephant and twice as large as the largest sauropod. However, the reasons for their enormous sizes or the possibility of still larger animals remains unclear. A deeper understanding of the evolutionary mechanisms shaping cetacean sizes would shed light on the role of energetic constraints in limiting species sizes [1], and the interaction of macroecological patterns [2] and macroevolutionary processes [3] in the oceans. It may also shed light on how long-term trends in species mass [4], [5], e.g., Cope’s rule, the empirically observed tendency for species masses to increase within a lineage over evolutionary time [6], [7], operate in marine environments.

**Figure 1:**
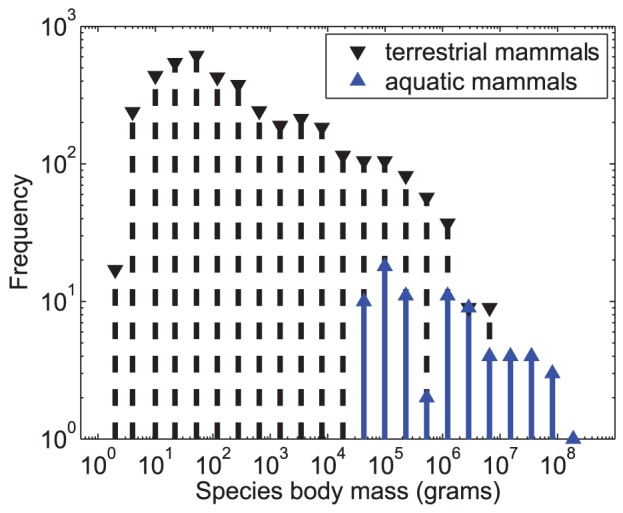
Terrestrial and fully aquatic mammal species mass distributions. Both show the canonical asymmetric pattern: the median size is flanked by a short left-tail down to a minimum viable size and a long right-tail out to a few extremely large species.

Many major animal clades, including mammals, birds, fish and insects, seem to exhibit a canonical pattern in the distribution of species masses [6], [8]–[10]. For example, the most common size of a terrestrial mammal is roughly 40 *g* (common Pacific Rat, *Rattus exulans*). Both larger and smaller species are much less common, but asymmetrically so: the largest species, like the extinct Imperial Mammoth (*Mammuthus imperator*, 10^7^ *g*), are orders of magnitude larger, while the smallest, like Remy’s Pygmy Shrew (*Suncus remyi*, 2 *g*), are only a little smaller (Fig. 1).

**Figure 2:**
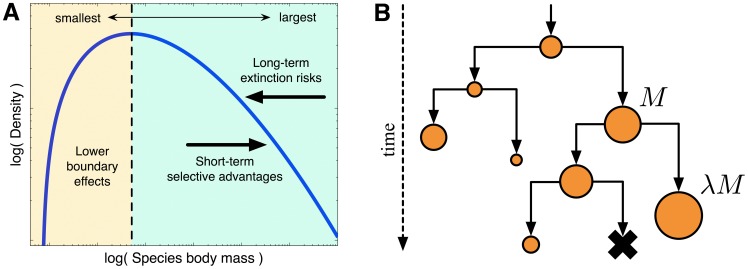
Characteristic species size pattern and cladogenetic diffusion model. (A) The characteristic distribution of species body sizes, observed in most major animal groups. Macroevolutionary tradeoffs between short-term selective advantages and long-term extinction risks, constrained by a minimum viable size 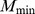, produce the distribution’s long right-tail. (B) Schematic illustrating the cladogenetic diffusion model of species body-size evolution: a descendant species’ mass is related to its ancestor’s size *M* by a random multiplicative factor 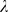. Species become extinct with a probability that grows slowly with *M*.

Both the precise shape and the origins of this ubiquitous pattern have long been a topic of ecological [11] and evolutionary [3], [12] interest. Recently, this pattern was shown to be a long-term evolutionary consequence when a minimum viable body size, e.g., from physiological or thermoregulatory limits [13], constrains a macroevolutionary tradeoff between short-term selective advantages [2] and long-term extinction risks from increased species size [10], [14] (Fig. 2a). Early versions of this model [3], [12] demonstrated that species size evolution in the presence of a fixed lower limit produces right-skewed distributions that are qualitatively similar to the empirical pattern. However, these models also predict an unending increase in the size of the largest species, without necessarily adding new species. The key missing mechanism is extinction risk, which empirically tends to increase with species body size [15], [16] and thereby limit the number and size of large species. In this way, the characteristic pattern in species sizes can be explained from simple macroevolutionary mechanisms: speciation, variation, extinction and a physiological minimum size.

Historically, the main alternative explanation assumed the existence of a taxon-specific energetically optimal body size [17]–[19]. At this size, species maximize their “reproductive power,” i.e., the rate at which they convert environmental resources into offspring. Dispersion away from this optimum size was interpreted as evidence of interspecific competition. However, this theory remains controversial and, among other reasons [8], contradicts strong evidence from the fossil record in the form of Cope’s rule, a general statistical tendency for descendant species to be larger than their ancestors [7], [10], and the fact that most species are not close to their group’s predicted optimal size.

Although the macroevolutionary tradeoff hypothesis has been quantitatively tested for extant terrestrial mammals [10] and birds [20], and its temporal dynamics have been shown to agree with the expansion of terrestrial mammals in the late Cretaceous and early Paleogene [14], it remains unknown precisely how general this hypothesis is. For instance, it is unknown whether it holds for subclades of Mammalia, for fully aquatic mammals (which have typically been omitted from previous analyses), for ectothermic species, etc.

We resolve several of these questions by testing the tradeoff theory’s ability to explain the observed body size distribution of cetaceans, the largest and most diverse marine mammal clade. Cetaceans are an ideal test case for the theory. First, Cetacea is a sufficiently speciose clade (77 extant species) to allow a quantitative comparison of predicted and observed distributions. Sirenia, the only other fully aquatic mammal clade, contains four extant species, which is too small for a productive comparison. Second, semiaquatic groups like Pinnipeds (seals and walruses) and Mustelids (otters) cannot be used to test the theory because they spend significant time on land, thus avoiding the hard thermoregulatory constraint assumed by the theory. Thus, by focusing on cetaceans, we provide a reasonable test of the theory. Third, fully aquatic mammals like cetaceans have typically been omitted in past studies because their marine habitat induces a different lower limit on mass than is seen in terrestrial mammals. As a result, it remains unknown whether the theory extends to all mammals, or only those in terrestrial environments. Finally, cetacean body masses do indeed exhibit the canonical right-skewed pattern (Fig. 1): the median size (356 kg, *Tursiops truncatus*) is close to the smallest (37.5 *kg*, *Pontoporia blainvillei*) but far from the largest (175,000 kg). This suggests that the theory may indeed hold for them.

Here, we test the strongest possible form of the macroevolutionary tradeoff theory for cetacean sizes. Instead of estimating model parameters from cetacean data, we combine parameters estimated from terrestrial mammals with a theoretically determined choice for the lower limit on cetacean species body mass. The resulting model has no tunable parameters by which to adjust its predicted distribution. In this way, we answer the question of how large a whale should be: if the predicted distribution agrees with the observed sizes, the same short-term versus long-term tradeoff that determines the sizes of terrestrial mammals also determines the sizes of whales.

We find that this zero-parameter model provides a highly accurate prediction of cetacean sizes. Thus, a single universal tradeoff mechanism appears to explain the body sizes of all mammal species, but this mechanism must obey the thermoregulatory limits imposed by the environment in which it unfolds. It is this one difference–thermoregulation in air for terrestrial mammals and in water for aquatic mammals–that explains the different locations of their respective body size distributions. Energetic constraints, while a popular historical explanation for sizes, seem to be only part of the puzzle for understanding the distribution of species sizes. Under this macroevolutionary mechanism, the size of the largest observed species is set by the tradeoff between the extinction probability at large sizes and the rate at which smaller species evolve to larger body masses, both of which may depend partly on energetic and ecological factors.

## Methods

Following Clauset and Erwin [10], we model the tradeoff hypothesis as constrained cladogenetic diffusion, which includes only simple stochastic processes like speciation, extinction, size variation, and a minimum viable size. Deviations between this null model and the observed sizes of species can be interpreted as the effects of processes omitted from the model, e.g., interspecific competition, environmental effects, etc. We then compare this model’s predictions to the observed sizes of all extant cetacean species.

### A Neutral Model of Species Body Sizes

Under the constrained diffusion model, a species of mass *M* produces descendant species with masses 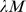 (Fig. 2b), where 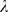 is a random variable summarizing the contributions from all sources of short-term selective effects on size [6], [12], including environmental gradients, interspecific competition and resource acquisition. For each speciation event, a new 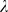 is drawn independently from a fixed distribution 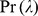. The interpretation of this model for variation in size down a lineage is that size-related short-term selection effects are uncorrelated across the clade. As a result, the distribution of sizes within the clade will evolve according to a diffusion process, and the trajectory of any particular lineage follows a kind of random walk [21], [22]. If the average size change between ancestors and descendants within a lineage is biased toward larger sizes (Cope’s rule), we have 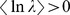 [7]. Anagenetic variation, or size variation between speciation events, need not be modeled separately as its impact may be absorbed into the 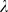 that describes the variation at the speciation event.

However, species may not take any size and thus the diffusion process is constrained. On the upper end, the probability of species extinction rises gently with increasing size [15], [16]. This size-dependency for extinction compactly summarize the systematic contributions from all sources to the overall extinction risk of larger-sized species, including larger energetic requirements [1], smaller species abundance [23], and longer generational times [24]. The net effect is a soft upper limit on species sizes, rather than a hard upper limit like those derived from energetic constraints alone [1]. Given a particular extinction risk curve, the number and size of the very largest species is determined by a macroevolutionary balance between the upward “pressure” of smaller-sized lineages migrating into the larger size ranges [25] and the downward extinction pressure of the increased extinction risk at those sizes.

On the lower end, endothermy imposes a minimum viable mass–a hard lower limit–that prohibits evolution toward ever smaller sizes. For terrestrial mammals and birds, this thermoregulatory minimum size is known to occur at roughly 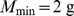 [13], [26], [27], below which a species’ convective heat loss in air is too high to maintain its internal temperature.

To extract a precise prediction of the species size distribution, we use a convection-diffusion-reaction formalization of the tradeoff theory [14], [20], which replaces the stochastic behavior of individual species and their lineages with a deterministic model of the relative density (fraction) of species at a given size. For analytic simplicity, we let the distribution of size changes 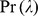 follow a log-normal distribution with parameters 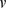 and 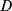, an assumption that is consistent with fossil data [10].

Let 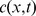 denote the density of species having mass 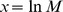 at time *t*. Under mild assumptions, the value 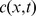 obeys the convection-diffusion-reaction equation in the continuum limit [28], [29]:
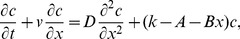
(1)where 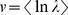 is the bias or average change in size from ancestor to descendent and 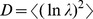 is the diffusion coefficient or the variance in size change. The expression 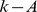 is the size-independent (background) net speciation rate, which sets the absolute scale of the mass frequencies, and *B* determines the strength and direction of a linear increase in extinction risk with the logarithm of species size.

In this model, the upper and lower size constraints guarantee the existence of a steady state distribution. To solve for its shape, we change variables 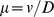, 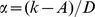, and 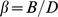, and require that the distribution go to zero at 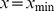. It can then be shown [14], [20] that the steady-state distribution of sizes *x* is
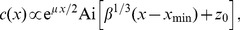
(2)where 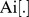 is the Airy function and 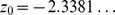 is the location of its first zero. The shape of this curve is fully determined by three model parameters: 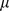, the normalized strength of Cope’s rule, 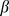, the normalized size-dependence of extinction risk, and 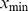, the logarithm of the minimum viable body size. To compare the sizes predicted by these macroevolutionary processes with those observed in real species, we must only choose values for the model parameters.

For terrestrial mammals, estimates for 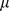 and 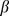 have previously been derived from fossil and extant data. The resulting size distribution accurately reproduces both the extant sizes of terrestrial mammals [10] and their expansion during the late Cretaceous and early Paleogene [14], [30]. Removing either the size-dependence of extinction risk or the minimum viable size produces unrealistic predictions [10].

The pelagic environments inhabited by cetaceans, however, impose distinct physiological, ecological and evolutionary challenges for endothermic mammals, and these are not reflected in the terrestrial model. One critical difference is the greater convective heat loss in water, which raises the minimum size of a competent aquatic endotherm. Thermoregulatory calculations and empirical data agree that this minimum size is roughly 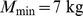 [13], [31], [32], about 3500 times larger than the minimum size imposed by thermoregulation in air.

### Testing the Tradeoff Hypothesis

A strong form of the macroevolutionary tradeoff hypothesis is to allow 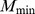 to vary based on whether a species lives on land or in water, but to assume universal values for 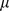 and 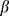, i.e., values that hold regardless of habitat. By using estimates of 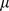 and 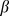 derived from terrestrial mammals alone, the model makes a prediction with no tunable parameters by which to adjust its fit to the observed cetacean sizes. This *ex ante* prediction either matches the data or it does not.

To test the prediction, we constructed a novel body size data set covering all 77 extant cetacean species, from 183 empirical size estimates [33]–[61]. Only plausibly independent, scientifically derived estimates were included. Mass ranges were converted to point estimates by taking their midpoint, unless a mean value was also provided. Subsequently, the mean value of all point estimates for a given species was used; this yielded an average of 2.4 measurements per species. Table S1 gives the mass estimates, primary source(s) and data curation comments.

We then evaluate the prediction’s accuracy in two ways. First, we construct a classic hypothesis test for this “zero parameter” prediction. Such a test assumes observations are generated by independent draws from a fixed distribution, when in fact real species sizes are correlated due to shared evolutionary history. As a result, the hypothesis test is inherently conservative. Failure to reject the null model would indicate strong support for the tradeoff theory and that deviations between the predicted and observed size distributions are not statistically significant. Second, we consider whether the largest observed species, the Blue Whale, is statistically unlikely under the model. Failure to reject the hypothesis here indicates strong support for the number and size of very large species being set primarily by the macroevolutionary tradeoff, rather than by energetics alone.

## Results

Previous analyses of terrestrial mammal data [14], [20] yielded 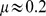, a slight tendency toward larger sizes within a lineage (Cope’s rule), and 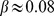, a weak tendency for extinction to increase with body size. Using these values and setting 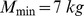 for fully aquatic species [31] completes the model parameterization under Eq. (2).

**Figure 3:**
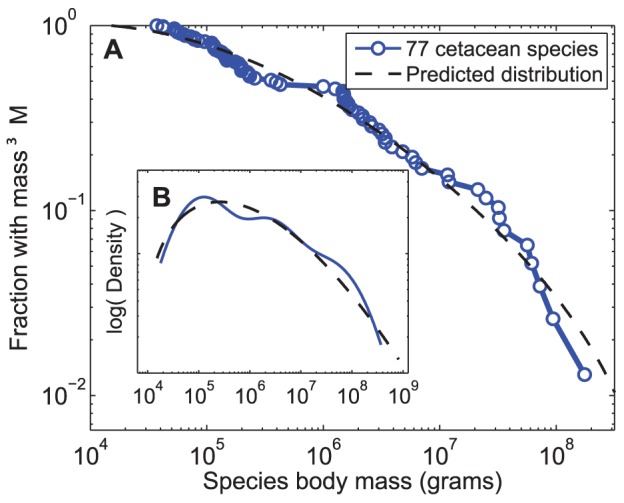
Comparison of data and model predictions. (A) *Ex ante* predicted cetacean sizes, from a cladogenetic model fitted to terrestrial mammals but with a pelagic 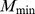 (see text), and empirical sizes of 77 extant cetacean species, as complementary cumulative distributions and as (B) smoothed probability densities.

Figure 3 shows the predicted and observed distributions. The predicted model’s statistical plausibility is determined by a standard two-tailed Kolmogorov-Smirnov hypothesis test, evaluated numerically. This produces a *p*-value of 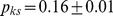, which exceeds the conventional threshold for rejecting the null hypothesis. This indicates that the distribution of observed masses for cetacean species are statistically indistinguishable from the masses predicted by the model. As a control on the statistical uncertainty in the values of 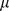 and 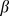, we conduct a second test in which we add a small amount of Normally distributed noise to these parameter values and recompute 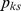 via Monte Carlo. This yields a slightly lower but still non-significant 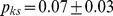.

We now consider whether the size of the largest observed cetacean species should be considered a statistical outlier under the model. The probability of observing at least one species with size at least as large as the Blue Whale at 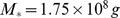 was computed as 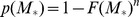 where 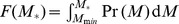 is the portion of the predicted distribution below 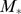 (the cdf) and 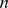 is the number of iid observations (extant species) drawn from 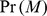. Taking fixed parameters yields 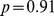, while simulating statistical uncertainty via Monte Carlo (as above) yields 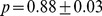, which is consistent with the fixed-parameter result.

These results imply that the observed sizes of whales are precisely what we would expect under a universal macroevolutionary tradeoff between short-term selective advantages and long-term extinction risks for increased size, unfolding under a constraint imposed by an environmentally-determined minimum viable size. This holds even for the enormous size of the Blue Whale, which is not statistically unlikely under this model. In fact, a species somewhat larger than the Blue Whale would also not be statistically unlikely, although no such species is known to have existed. Mathematically, the expected maximum size lies at 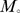, the solution to the equation 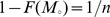. Using our cetacean model, we find this value to be roughly 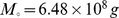, or about 3.7 times larger than the Blue Whale.

As a robustness check on our results, we test the assumption that 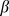 takes a universal value for all mammals. Specifically, we hold 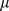 fixed at the terrestrial value and estimate 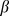 by fitting Eq. (2) to the observed cetacean sizes. This procedure yields 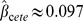, which is close to the terrestrial mammal value of 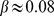 [14], [20] and supports our assumption of a universal extinction risk curve for mammalian evolution. Furthermore, using this fitted value in the cetacean model, instead of the terrestrial value, would only reduce the statistical differences between the model and the data, and thus would not change our overall results. A similar check on the universality of 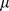 cannot be conducted at this time. The value of 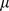 is most reliably estimated from comprehensive data on fossil species sizes [20], which is not currently available for cetaceans.

## Discussion

It is remarkable that the predicted distribution, which has no tunable parameters, is statistically indistinguishable from the observed sizes of cetaceans. Rarely in biological systems are the predictions of mathematical models so unambiguous and rarely are they upheld so clearly when compared to empirical data. This result thus strongly supports the hypothesis that both terrestrial and aquatic mammal sizes are shaped by a single universal macroevolutionary tradeoff between short-term advantages and long-term extinction risks of increased size, but which is constrained by a habitat-specific lower limit on size.

The only difference between our terrestrial and aquatic mammal tradeoff models is a larger minimum size for cetaceans, due greater convective heat loss in water. The macroevolutionary consequence is to shift upward the entire canonical species size distribution, pushing its right tail out into size ranges inaccessible to terrestrial mammals and producing giants like the Blue Whale. In this way we answer our motivating question of how large should whales be: they are as a group exactly as large as we should expect for mammals evolving under the thermoregulatory constraint of fully aquatic life. And, if we were given the first archaeocete’s size, species counts over geological time and the model diffusion rate, the model would allow us to predict when a species of a given size should first have appeared.

The lower limit on size for a fully aquatic species would also have played a significant role over the long history of the Mammalia clade. From their emergence roughly 210 Ma to roughly 60 Ma, mammals were typically small-bodied [62], with few or no species exceeding 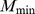 for pelagic niches. Thus, aquatic lifestyles and the enormous body sizes associated with them would have been effectively inaccessible. In short, whales could not have evolved during this period. It was only in the late Cretaceous and early Paleogene, when the terrestrial mammal size distribution began expanding [14], [30] that there were sufficient numbers of species above the threshold for a transition into pelagic habitats to be possible.

It is interesting to note that almost immediately after the terrestrial size distribution extended beyond the pelagic minimum, mammals did indeed invaded the oceans. This coincidence suggests a kind of body-size mediated ecological release, in which the expansion of the species size distribution enabled a dramatic and qualitative change in the large-scale occupation of ecological niches. For this reason, the historical timing of when mammals returned to the oceans is explained by the timing of late Cretaceous and early Paleogene expansion relative to the particular size required for a fully aquatic lifestyle.

On the upper end of sizes, some past work has considered the possibility of maximum species sizes due to energetic constraints [1], [13]. For instance, in the case of powered flight, decreasing metabolic power per unit mass effectively makes it difficult for birds above 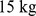 to generate sufficient power for flapping flight [13]. Of course, flightless birds like the ostrich (at roughly 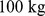) have circumvented this constraint by abandoning flight altogether. In the case of cetaceans, recent work suggests a similar decreasing power delivery per unit mass during lunge feeding in large mysticetes [63], [64]. This tendency suggests a maximum species size caused by the increased difficulty faced by very large whales in satisfying their energetic requirements. In principle, however, whales may be able to circumvent this limit by changing their feeding behavior or food source [64].

Although it is reasonable to argue that whales cannot evolve to arbitrarily large sizes, it remains unclear whether a genuine maximum size from energetic constraints is low enough to impact the observed distribution of sizes. Our results suggest that there is no statistical evidence for such a limit in the vicinity of the Blue Whale’s mass at 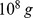, as we achieve statistical indistinguishability without an explicit limit. In fact, a slightly larger species would also not be statistically unlikely under the model, suggesting that the Blue Whale’s size may arise more from its particular energetically-suboptimal lunging strategy [64] than from a fundamental limit on all possible cetaceans.

The macroevolutionary tradeoff theory does produce a general upper limit on size: the largest observed species occurs at a size close to where the net speciation rate effectively falls to zero, which is a finite value for any finite-sized clade. With 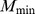 fixed by the environment, the precise location of this point depends on the rate at which smaller-bodied species evolve to larger sizes (captured by the model parameter 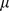), the rate at which extinction eliminates them (captured by 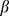), and evolutionary fluctuations. This type of macroevolutionary turnover at the largest sizes is known to have occurred repeatedly in North American canids [25]. The tradeoff theory implies that the pattern is ubiquitous, and should also occur in cetaceans.

At the macroevolutionary level of analysis considered here, the effects of energetics, population size, generation time, interspecific competition, morphology, geography, climate, etc. are all implicitly captured by the structure and parameters of the diffusion and extinction processes. The highly abstract nature of this theory does not undermine the importance of these factors for explaining the sizes of specific species in specific environments. It merely implies that across the clade and across large spatial and temporal scales, these factors collectively exert gentle macroevolutionary pressures that can be compactly summarized by a constrained diffusion model. For investigating species sizes within specific clades, the tradeoff hypothesis should be viewed as a kind of “neutral” model. Statistically significant deviations imply the presence of non-neutral evolutionary or ecological processes. In the same way, changes in model parameter values over deep time may indicate broad-scale, non-stationary processes like climate change or clade-level ecological competition, as between mammals and dinosaurs prior to the K-Pg event.

In closing, we point out that this tradeoff between short-term advantages and long-term risks for increased size is entirely general. To date, however, it has only be tested on endotherms like mammals and birds. If the theory also holds for other major clades, such as aquatic tetrapod groups like icthyosaurs, plesiosaurs and turtles, groups like dinosaurs, fish and foraminifera, or subclades within these groups, it would have major implications for our understanding of macroevolution. A broad examination of minimum viable sizes and size-dependent extinction risks across groups and geologic time would thus better elucidate the role of these mechanisms in shaping the trajectory of species sizes throughout the history of life.

## Supporting Information

Table S1 **Body mass estimates of extant cetacean species.** 183 mass estimates across 77 extant cetacean species, with primary source (reference) and data curation notes.
(PDF)

## References

[1] McNab BK (2009) Resources and energetics determine dinosaur maximal size. Proc Natl Acad Sci (USA) 106: 12184–12188. 19581600 10.1073/pnas.0904000106 PMC2715483

[2] Brown JH (1995) Macroecology. Chicago: University of Chicago Press.

[3] Stanley SM (1975) A theory of evolution above the species level. Proc Natl Acad Sci (USA) 72: 646–650. 1054846 10.1073/pnas.72.2.646 PMC432371

[4] Alroy J (2000) Understanding the dynamics of trends within evolving lineages. Paleobiology 26: 319–329.

[5] Alroy J (2000) New methods for quantifying macroevolutionary patterns and processes. Paleobiology 26: 707–733.

[6] Stanley SM (1973) An explanation for Cope’s Rule. Evolution 27: 1–26. 28563664 10.1111/j.1558-5646.1973.tb05912.x

[7] Alroy J (1998) Cope’s rule and the dynamics of body mass evolution in North American fossil mammals. Science 280: 731–734. 9563948 10.1126/science.280.5364.731

[8] Koz lowski J, Gawelczyk AT (2002) Why are species’ body size distributions usually skewed to the right? Functional Ecology 16: 419–432.

[9] Allen CR, Garmestani AS, Havlicek TD, Marquet PA, Peterson GD, et al (2006) Patterns in body mass distributions: Sifting among alternative hypotheses. Ecology Letters 9: 630–643. 16643307 10.1111/j.1461-0248.2006.00902.x

[10] Clauset A, Erwin DH (2008) The evolution and distribution of species body size. Science 321: 399–401. 18635801 10.1126/science.1157534

[11] Smith FA, Lyons SK (2011) How big should a mammal be? A macroecological look at mammalian body size over space and time. Phil Trans R Soc B 366: 2364–2378. 21768152 10.1098/rstb.2011.0067 PMC3130437

[12] McShea DW (1994) Mechanisms of large-scale evolutionary trends. Evolution 48: 1747–1763. 28565153 10.1111/j.1558-5646.1994.tb02211.x

[13] Ahlborn BK (2000) Thermodynamic limits of body dimension of warm blooded animals. J Non-Equilib Thermodyn 25: 87–102.

[14] Clauset A, Redner S (2009) Evolutionary model of species body mass diversification. Physical Review Letters 102: 038103. 19257399 10.1103/PhysRevLett.102.038103

[15] Liow LH, Fortelius M, Bingham E, Lintulaakso K, Mannila H, et al (2008) Higher origination and extinction rates in larger mammals. Proc Natl Acad Sci (USA) 105: 6097–6102. 18417455 10.1073/pnas.0709763105 PMC2329699

[16] Davidson AD, Boyer AG, Kim H, Pompa-Mansilla S, Hamilton MJ, et al (2012) Drivers and hotspots of extinction risk in marine mammals. Proc Natl Acad Sci (USA) 109: 3395–3400. 22308490 10.1073/pnas.1121469109 PMC3295301

[17] Lomolino M (1985) Body size of mammals on islands: the island rule re-examined. American Naturalist 125: 310–316.

[18] Sebens KP (1987) The ecology of indeterminate growth in animals. Annual Review of Ecology and Systematics 18: 371–407.

[19] Brown JH, Marquet PA, Taper ML (1996) Darwinian fitness and reproductive power: Reply to koz lowski. American Naturalist 147: 1092–1097.

[20] Clauset A, Schwab DJ, Redner S (2009) How many species have mass *M*? American Naturalist 173: 256–263. 10.1086/595760 19090772

[21] Raup DM (1977) Probabilistic models in evolutionary paleobiology: A random walk through the fossil record produces some surprising results. American Scientist 65: 50–57. 842931

[22] Hunt G (2007) The relative importance of directional change, random walks, and stasis in the evolution of fossil lineages. Proc Natl Acad Sci (USA) 104: 18404–18408. 18003931 10.1073/pnas.0704088104 PMC2141789

[23] White EP, Morgan Ernest SK, Kerkhoff AJ, Enquist BJ (2007) Relationships between body size and abundance in ecology. Trends in Ecology and Evolution 22: 323–330. 17399851 10.1016/j.tree.2007.03.007

[24] Martin AP, Palumbi SR (1993) Body size, metabolic rate, generation time, and the molecular clock. Proc Natl Acad Sci (USA) 90: 4087–4091. 8483925 10.1073/pnas.90.9.4087 PMC46451

[25] Van Valkenburgh B, Wang X, Damuth J (2004) Cope’s rule, hypercarnivory, and extinction in North American canids. Science 306: 101–104. 15459388 10.1126/science.1102417

[26] Pearson OP (1948) Metabolism of small mammals, with remarks on the lower limit of mammalian size. Science 108: 44. 17739234 10.1126/science.108.2793.44

[27] West GB, Woodruff WH, Brown JH (2002) Allometric scaling of metabolic rate from molecules and mitochondria to cells and mammals. Proc Natl Acad Sci (USA) 99: 2473–2478. 11875197 10.1073/pnas.012579799 PMC128563

[28] Berg HC (1993) Random Walks in Biology. Princeton University Press.

[29] Krapivsky PL, Redner S, Ben-Naim E (2010) A Kinetic View of Statistical Physics. Cambridge University Press.

[30] Wilson GP, Evans AR, Corfe IJ, Smits PD, Fortelius M, et al (2012) Adaptive radiation of multituberculate mammals before the extinction of dinosaurs. Nature 483: 457–460. 22419156 10.1038/nature10880

[31] Downhower JF, Blumer LS (1988) Calculating just how small a whale can be. Nature 335: 675.3173490

[32] Ahlborn BK, Blake RW (1999) Lower size limit of aquatic mammals. Am J Phys 67: 920–922.

[33] Long CA (1968) An analysis of patterns of variation in some representative Mammalia. Part I. A review of estimates of variability in selected measurements. Transactions of the Kansas Academy of Science (1903–) 71: 201–227. 5672063

[34] Reeves RR, Tracey S (1980) Monodon monoceros. Mammalian Species 127: 1–7.

[35] Mead JG, Walker WA, Houck WJ, Smithsonian Institution (1982) Biological Observations on *Mesoplodon carlhubbsi* (Cetacea, Ziphiidae). Smithsonian contributions to zoology. Smithsonian Institution Press.

[36] Nagorsen D (1985) Kogia simus. Mammalian Species 239: 1–6.

[37] Stewart BE, Stewart REA (1989) Delphinapterus leucas. Mammalian Species 336: 1–8.

[38] Best RC, da Silva VMF (1993) Inia geoffrensis. Mammalian Species 426: 1–8.

[39] Jefferson TA, Leatherwood S, Webber MA (1993) FAO species identification guide. Marine mammals of the world. Food and Agriculture Organization of the United Nations.

[40] Jefferson TA, Newcomer MW (1993) Lissodelphis borealis. Mammalian Species 425: 1–6.

[41] Stacey PJ, Leatherwood S, Baird RW (1994) Pseudorca crassidens. Mammalian Species 456: 1–6.

[42] Jefferson TA, Leatherwood S (1994) Right whale dolphins *Lissodelphis borealis* (Peale, 1848) and *Lissodelphis peronii* (Lacëpéde, 1804). Handbook of Marine Mammals 5: 335–362.

[43] Jefferson TA, Leatherwood S (1994) Lagenodelphis hosei. Mammalian Species 470: 1–5.

[44] Newcomer MW, Jefferson TA, Brownell, Jr RL (1996) Lissodelphis peronii. Mammalian Species 531: 1–5.

[45] Jefferson TA, Barros NB (1997) Peponocephala electra. Mammalian Species 553: 1–6.

[46] Uhen MD, Fordyce RE, Barnes LG (1998) Odontoceti. In: Janis CM, Gunnell GF, Uhen MD, editors, Evolution of Tertiary Mammals of North American Volume 2: Small Mammals, Xenarthrans, and Marine Mammals, Cambridge University Press. 566–606.

[47] Uhen MD, Fordyce RE, Barnes LG (1998) Mysticeti. In: Janis CM, Gunnell GF, Uhen MD, editors, Evolution of Tertiary Mammals of North American Volume 2: Small Mammals, Xenarthrans, and Marine Mammals, Cambridge University Press. 607–628.

[48] Perrin WF (1998) Stenella longirostris. Mammalian Species 599: 1–7.

[49] Clapham PJ, Mead JG (1999) Megaptera novaeangliae. Mammalian Species 604: 1–9.

[50] Cranford TW (1999) The sperm whale’s nose: Sexual selection on a grand scale? Marine Mammal Science 15: 1133–1157.

[51] Stacey PJ, Arnold PW (1999) Orcaella brevirostris. Mammalian Species 616: 1–8.

[52] Jefferson TA, Karczmarski L (2001) Sousa chinensis. Mammalian Species 655: 1–9.

[53] Perrin WF (2001) Stenella attenuata. Mammalian Species 683: 1–8.

[54] Perrin WF (2002) Stenella frontalis. Mammalian Species 702: 1–6.

[55] Smith FA, Lyons SK, Ernest SKM, Jones KE, Kaufman DM, et al (2003) Body mass of Late Quaternary mammals. Ecology 84: 3403.

[56] Jefferson TA, Curry BE (2003) Stenella clymene. Mammalian Species 726: 1–5.

[57] Perrin WF, Zubtsova GE, Kuz’min AA (2004) Partial Catalog of Cetacean Osteological Specimens in Russian Museums. NOAA Technical Memorandum NMFS. NOM-TM-NMFS-SWFSC-364.

[58] Culik BM (2004) Review of Small Cetaceans. Bonn, Germany: Conservation of Migratory Species of Wild Animals Secretariat. Marine Mammal Action Plan/Regional Seas Report and Studies no.177.

[59] Van Waerebeek K, Barnett L, Camara A, Cham A, Diallo M, et al (2004) Distribution, status, and biology of the atlantic humpback dolphin, Sousa teuszii (Kükenthal, 1892). Aquatic Mammals 30: 56–83.

[60] Jefferson TA, Hung SK (2004) Neophocaena phocaenoides. Mammalian Species 746: 1–12.

[61] Borsa P (2006) Marine mammal strandings in the New Caledonia region, Southwest Pacific. Comptes Rendus Biologies 329: 277–288. 16644500 10.1016/j.crvi.2006.01.004

[62] Luo ZX (2007) Transformation and diversification in early mammal evolution. Nature 450: 1011–1019. 18075580 10.1038/nature06277

[63] Goldbogen JA, Calambokidis J, Croll DA, McKenna MF, Oleson E, et al (2012) Scaling of lungefeeding performance in rorqual whales: Mass-specific energy expenditure increases with body size and progressively limits diving capacity. Functional Ecology 26: 216–226.

[64] Potvin J, Goldbogen JA, Shadwick RE (2012) Metabolic expenditures of lunge feeding rorquals across scale: Implications for the evolution of filter feeding and the limits to maximum body size. PLOS ONE 7: e44854. 23024769 10.1371/journal.pone.0044854 PMC3443106

