# Supplementary material for: How Large Should Whales Be?
Source: PLoS One. 2013 Jan 10;8(1):e53967. doi: 10.1371/journal.pone.0053967 (PMC3546790; doi:10.1371/journal.pone.0053967)
Supplement: Table S1 — Body mass estimates of extant cetacean species. 183 mass estimates across 77 extant cetacean species, with primary source (reference) and data curation notes. (PDF) [file Table_S1.pdf]

group	family	species	mass_kg	primary source (reference)	notes
Mysticeti	Balaenidae	Balaena mysticetus	100000	Smith_etal_2003	.
Mysticeti	Balaenidae	Balaena mysticetus	87500	Jefferson_Leatherwood_Webber_1993	.
Mysticeti	Balaenidae	Eubalaena australis	23000	Smith_etal_2003	.
Mysticeti	Balaenidae	Eubalaena australis	100000	Jefferson_Leatherwood_Webber_1993	.
Mysticeti	Balaenidae	Eubalaena glacialis	23000	Smith_etal_2003	.
Mysticeti	Balaenidae	Eubalaena glacialis	90000	Jefferson_Leatherwood_Webber_1993	.
Mysticeti	Balaenopteridae	Balaenoptera acutorostrata	10000	Smith_etal_2003	.
Mysticeti	Balaenopteridae	Balaenoptera acutorostrata	14000	Jefferson_Leatherwood_Webber_1993, Perrin_Zubtsova_Kuzmin_2004	.
Mysticeti	Balaenopteridae	Balaenoptera borealis	20000	Smith_etal_2003	.
Mysticeti	Balaenopteridae	Balaenoptera borealis	30000	Jefferson_Leatherwood_Webber_1993, Long_1968	.
Mysticeti	Balaenopteridae	Balaenoptera edeni	20000	Smith_etal_2003	.
Mysticeti	Balaenopteridae	Balaenoptera edeni	22500	Jefferson_Leatherwood_Webber_1993	.
Mysticeti	Balaenopteridae	Balaenoptera musculus	190000	Smith_etal_2003	.
Mysticeti	Balaenopteridae	Balaenoptera musculus	160000	Jefferson_Leatherwood_Webber_1993, Morton_ed_1997	.
Mysticeti	Balaenopteridae	Balaenoptera physalus	70000	Smith_etal_2003	.
Mysticeti	Balaenopteridae	Balaenoptera physalus	75000	Jefferson_Leatherwood_Webber_1993, Uhen_Fordyce_Barnes_1998_inJanisGunnellUhen	.
Mysticeti	Balaenopteridae	Megaptera novaeangliae	30000	Smith_etal_2003	.
Mysticeti	Balaenopteridae	Megaptera novaeangliae	35000	Jefferson_Leatherwood_Webber_1993, Clapham_Mead_1999, Uhen_Fordyce_Barnes_1998_inJanisGunnellUhen	.
Mysticeti	Eschrichtiidae	Eschrichtius robustus	28500	Smith_etal_2003	.
Mysticeti	Eschrichtiidae	Eschrichtius robustus	35000	Jefferson_Leatherwood_Webber_1993, Uhen_Fordyce_Barnes_1998_inJanisGunnellUhen	.
Mysticeti	Neobalaenidae	Caperea marginata	3200	Jefferson_Leatherwood_Webber_1993, Perrin_Zubtsova_Kuzmin_2004	note Smith_etal_2003 give a mass 10x as large, so we exclude it as being erroneous
Odontoceti	Delphinidae	Cephalorhynchus commersonii	72.4	Smith_etal_2003	.
Odontoceti	Delphinidae	Cephalorhynchus commersonii	76	Jefferson_Leatherwood_Webber_1993	.
Odontoceti	Delphinidae	Cephalorhynchus commersonii	86	Culik_2004	.
Odontoceti	Delphinidae	Cephalorhynchus eutropia	45	Smith_etal_2003	.
Odontoceti	Delphinidae	Cephalorhynchus eutropia	63	Jefferson_Leatherwood_Webber_1993	.
Odontoceti	Delphinidae	Cephalorhynchus eutropia	60	Culik_2004	.
Odontoceti	Delphinidae	Cephalorhynchus heavisidii	40	Smith_etal_2003	.
Odontoceti	Delphinidae	Cephalorhynchus heavisidii	65	Culik_2004	.
Odontoceti	Delphinidae	Cephalorhynchus hectori	50	Smith_etal_2003	.
Odontoceti	Delphinidae	Cephalorhynchus hectori	57	Jefferson_Leatherwood_Webber_1993	.
Odontoceti	Delphinidae	Delphinus delphis	80	Smith_etal_2003	.
Odontoceti	Delphinidae	Delphinus delphis	135	Jefferson_Leatherwood_Webber_1993	.
Odontoceti	Delphinidae	Delphinus delphis	200	Culik_2004	.
Odontoceti	Delphinidae	Feresa attenuata	170	Smith_etal_2003	.
Odontoceti	Delphinidae	Feresa attenuata	225	Jefferson_Leatherwood_Webber_1993	.
Odontoceti	Delphinidae	Globicephala macrorhynchus	726	Smith_etal_2003	.
Odontoceti	Delphinidae	Globicephala macrorhynchus	3600	Jefferson_Leatherwood_Webber_1993	.
Odontoceti	Delphinidae	Globicephala melas	800	Smith_etal_2003	.
Odontoceti	Delphinidae	Globicephala melas	2000	Jefferson_Leatherwood_Webber_1993	.
Odontoceti	Delphinidae	Globicephala melas	1600	Perrin_Zubtsova_Kuzmin_2004	.
Odontoceti	Delphinidae	Grampus griseus	387.5	Smith_etal_2003	.
Odontoceti	Delphinidae	Grampus griseus	400	Jefferson_Leatherwood_Webber_1993	.
Odontoceti	Delphinidae	Lagenodelphis hosei	164	Smith_etal_2003	.
Odontoceti	Delphinidae	Lagenodelphis hosei	210	Jefferson_Leatherwood_Webber_1993	.
Odontoceti	Delphinidae	Lagenodelphis hosei	210	Culik_2004	.
Odontoceti	Delphinidae	Lagenodelphis hosei	209	Jefferson_Leatherwood_1994	.
Odontoceti	Delphinidae	Lagenorhynchus acutus	182	Smith_etal_2003	.
Odontoceti	Delphinidae	Lagenorhynchus acutus	208.5	Jefferson_Leatherwood_Webber_1993	.
Odontoceti	Delphinidae	Lagenorhynchus acutus	205	Culik_2004	.
Odontoceti	Delphinidae	Lagenorhynchus albirostris	180	Smith_etal_2003	.
Odontoceti	Delphinidae	Lagenorhynchus albirostris	265	Culik_2004	.
Odontoceti	Delphinidae	Lagenorhynchus australis	120	Smith_etal_2003	.

Odontoceti	Delphinidae	Lagenorhynchus australis	115	Jefferson_Leatherwood_Webber_1993	.
Odontoceti	Delphinidae	Lagenorhynchus australis	115	Culik_2004	.
Odontoceti	Delphinidae	Lagenorhynchus cruciger	110	Smith_etal_2003	.
Odontoceti	Delphinidae	Lagenorhynchus obliquidens	120	Smith_etal_2003	.
Odontoceti	Delphinidae	Lagenorhynchus obliquidens	180	Jefferson_Leatherwood_Webber_1993	.
Odontoceti	Delphinidae	Lagenorhynchus obliquidens	82.5	Culik_2004	.
Odontoceti	Delphinidae	Lagenorhynchus obscurus	127.5	Smith_etal_2003	.
Odontoceti	Delphinidae	Lagenorhynchus obscurus	60	Jefferson_Leatherwood_Webber_1993	.
Odontoceti	Delphinidae	Lagenorhynchus obscurus	100	Culik_2004	.
Odontoceti	Delphinidae	Lissodelphis borealis	113	Smith_etal_2003	.
Odontoceti	Delphinidae	Lissodelphis borealis	115	Jefferson_Leatherwood_Webber_1993	.
Odontoceti	Delphinidae	Lissodelphis borealis	116	Culik_2004	.
Odontoceti	Delphinidae	Lissodelphis borealis	113	Jefferson_Newcomer_1993	.
Odontoceti	Delphinidae	Lissodelphis peronii	116	Smith_etal_2003	.
Odontoceti	Delphinidae	Lissodelphis peronii	116	Jefferson_Leatherwood_Webber_1993	Culik_2004 repeats these measurements and so we omit them
Odontoceti	Delphinidae	Lissodelphis peronii	116	Newcomer_Jefferson_Brownell_1996	.
Odontoceti	Delphinidae	Orcaella brevirostris	190	Smith_etal_2003	.
Odontoceti	Delphinidae	Orcaella brevirostris	122.5	Culik_2004	.
Odontoceti	Delphinidae	Orcaella brevirostris	123.5	Stacey_Arnold_1999	.
Odontoceti	Delphinidae	Orcinus orca	4300	Smith_etal_2003	.
Odontoceti	Delphinidae	Orcinus orca	8750	Jefferson_Leatherwood_Webber_1993	.
Odontoceti	Delphinidae	Orcinus orca	4685	Culik_2004	.
Odontoceti	Delphinidae	Orcinus orca	7050	Perrin_Zubtsova_Kuzmin_2004	reported mass mean of 2 specimens
Odontoceti	Delphinidae	Peponocephala electra	208	Smith_etal_2003	.
Odontoceti	Delphinidae	Peponocephala electra	275	Jefferson_Leatherwood_Webber_1993	.
Odontoceti	Delphinidae	Peponocephala electra	228	Culik_2004	.
Odontoceti	Delphinidae	Peponocephala electra	208	Jefferson_Barros_1997	.
Odontoceti	Delphinidae	Pseudorca crassidens	1360	Smith_etal_2003	.
Odontoceti	Delphinidae	Pseudorca crassidens	2000	Jefferson_Leatherwood_Webber_1993	.
Odontoceti	Delphinidae	Pseudorca crassidens	1360	Stacey_Leatherwood_1994	.
Odontoceti	Delphinidae	Sotalia fluviatilis	44	Smith_etal_2003	.
Odontoceti	Delphinidae	Sotalia fluviatilis	40	Jefferson_Leatherwood_Webber_1993	.
Odontoceti	Delphinidae	Sousa chinensis	265	Smith_etal_2003	.
Odontoceti	Delphinidae	Sousa chinensis	284	Jefferson_Leatherwood_Webber_1993	.
Odontoceti	Delphinidae	Sousa chinensis	215	Culik_2004	.
Odontoceti	Delphinidae	Sousa chinensis	265	Jefferson_Karczmarksi_2001	.
Odontoceti	Delphinidae	Sousa teuszii	100	Smith_etal_2003	.
Odontoceti	Delphinidae	Sousa teuszii	284	Jefferson_Leatherwood_Webber_1993	.
Odontoceti	Delphinidae	Sousa teuszii	215	Culik_2004	.
Odontoceti	Delphinidae	Sousa teuszii	166	Waerebeek_etal_2004	.
Odontoceti	Delphinidae	Stenella attenuata	120	Jefferson_Leatherwood_Webber_1993	estimate from Smith_etal_2003 is less than 1/2 of the values reported elsewhere and is thus omitted
Odontoceti	Delphinidae	Stenella attenuata	119	Culik_2004,	.
Odontoceti	Delphinidae	Stenella attenuata	119	Perrin_2001	.
Odontoceti	Delphinidae	Stenella clymene	68	Smith_etal_2003	.
Odontoceti	Delphinidae	Stenella clymene	85	Jefferson_Leatherwood_Webber_1993	.
Odontoceti	Delphinidae	Stenella clymene	80	Culik_2004	.
Odontoceti	Delphinidae	Stenella clymene	80	Jefferson_Curry_2003	.
Odontoceti	Delphinidae	Stenella coeruleoalba	135.9	Smith_etal_2003	.
Odontoceti	Delphinidae	Stenella coeruleoalba	156	Jefferson_Leatherwood_Webber_1993	.
Odontoceti	Delphinidae	Stenella coeruleoalba	156	Culik_2004	.
Odontoceti	Delphinidae	Stenella frontalis	110	Smith_etal_2003	.
Odontoceti	Delphinidae	Stenella frontalis	143	Jefferson_Leatherwood_Webber_1993	.
Odontoceti	Delphinidae	Stenella frontalis	143	Culik_2004	.
Odontoceti	Delphinidae	Stenella frontalis	140	Perrin_2002	.
Odontoceti	Delphinidae	Stenella longirostris	50.5	Smith_etal_2003	.
Odontoceti	Delphinidae	Stenella longirostris	77	Jefferson_Leatherwood_Webber_1993	.
Odontoceti	Delphinidae	Stenella longirostris	50.5	Perrin_1998	Culik_2004 repeats L and M measurements of Perrin_1998 and so are omitted
Odontoceti	Delphinidae	Steno bredanensis	130	Smith_etal_2003	.
Odontoceti	Delphinidae	Steno bredanensis	150	Jefferson_Leatherwood_Webber_1993	.
Odontoceti	Delphinidae	Steno bredanensis	155	Culik_2004	.
Odontoceti	Delphinidae	Tursiops truncatus	175	Smith_etal_2003	.

Odontoceti	Delphinidae	Tursiops truncatus	650	Jefferson_Leatherwood_Webber_1993	reported mass omitted (being more than 2x the other reported means)
Odontoceti	Delphinidae	Tursiops truncatus	242	Culik_2004	.
Odontoceti	Monodontidae	Delphinapterus leucas	1360	Smith_etal_2003	.
Odontoceti	Monodontidae	Delphinapterus leucas	1500	Steward_Steward_1989, Uhen_Fordyce_Barnes_1998_inJanisGunnellUhen	.
Odontoceti	Monodontidae	Delphinapterus leucas	1500	Culik_2004	.
Odontoceti	Monodontidae	Delphinapterus leucas	1600	Jefferson_Leatherwood_Webber_1993	.
Odontoceti	Monodontidae	Monodon monoceros	900	Smith_etal_2003	.
Odontoceti	Monodontidae	Monodon monoceros	1600	Jefferson_Leatherwood_Webber_1993, Reeves_Tracey_1980	.
Odontoceti	Monodontidae	Monodon monoceros	1300	Culik_2004	.
Odontoceti	Phocoenidae	Australophocaena dioptrica	65	Smith_etal_2003	.
Odontoceti	Phocoenidae	Neophocaena phocaenoides	32.5	Smith_etal_2003	.
Odontoceti	Phocoenidae	Neophocaena phocaenoides	85	Culik_2004	.
Odontoceti	Phocoenidae	Neophocaena phocaenoides	71.8	Jefferson_Hung_2004	.
Odontoceti	Phocoenidae	Phocoena phocoena	52.5	Smith_etal_2003	.
Odontoceti	Phocoenidae	Phocoena phocoena	57.5	Jefferson_Leatherwood_Webber_1993	.
Odontoceti	Phocoenidae	Phocoena phocoena	55	Culik_2004	.
Odontoceti	Phocoenidae	Phocoena sinus	42.5	Smith_etal_2003	.
Odontoceti	Phocoenidae	Phocoena spinipinnis	60	Smith_etal_2003	.
Odontoceti	Phocoenidae	Phocoena spinipinnis	85	Jefferson_Leatherwood_Webber_1993	.
Odontoceti	Phocoenidae	Phocoenoides dalli	102.5	Smith_etal_2003	.
Odontoceti	Phocoenidae	Phocoenoides dalli	200	Jefferson_Leatherwood_Webber_1993	.
Odontoceti	Phocoenidae	Phocoenoides dalli	200	Culik_2004	.
Odontoceti	Phocoenidae	Phocoenoides dalli	200	Jefferson_1988	.
Odontoceti	Physeteridae	Kogia breviceps	431.5	Culik_2004, Borsa_2006, Uhen_Fordyce_Barnes_1998_inJanisGunnellUhen	.
Odontoceti	Physeteridae	Kogia breviceps	450	Culik_2004	.
Odontoceti	Physeteridae	Kogia breviceps	400	Borsa_2006, Jefferson_Leatherwood_Webber_1993, Uhen_Fordyce_Barnes_1998_inJanisGunnellUhen	.
Odontoceti	Physeteridae	Kogia simus	183.1	Smith_etal_2003	.
Odontoceti	Physeteridae	Kogia simus	270	Nagorsen_1985	.
Odontoceti	Physeteridae	Kogia simus	270	Culik_2004	mass quoted as 2702 kg, but this is too big by an order of magnitude. Assumed to be 270.2kg
Odontoceti	Physeteridae	Kogia simus	210	Jefferson_Leatherwood_Webber_1993	.
Odontoceti	Physeteridae	Physeter catodon	14025	Smith_etal_2003	.
Odontoceti	Physeteridae	Physeter catodon	57000	Jefferson_Leatherwood_Webber_1993, Cranford_1999	.
Odontoceti	Platanistidae	Inia geoffrensis	129.25	Smith_etal_2003	.
Odontoceti	Platanistidae	Inia geoffrensis	160	Jefferson_Leatherwood_Webber_1993	.
Odontoceti	Platanistidae	Inia geoffrensis	167.5	Culik_2004	.
Odontoceti	Platanistidae	Inia geoffrensis	129.25	Best_Silva_1993	.
Odontoceti	Platanistidae	Lipotes vexillifer	187.5	Jefferson_Leatherwood_Webber_1993	mass estimate from Smith_etal_2003 is less than 1/2 estimated range here, and is thus omitted
Odontoceti	Platanistidae	Platanista gangetica	115	Smith_etal_2003	.
Odontoceti	Platanistidae	Platanista gangetica	108	Jefferson_Leatherwood_Webber_1993	.
Odontoceti	Platanistidae	Platanista minor	83.9146	Smith_etal_2003	.
Odontoceti	Platanistidae	Pontoporia blainvillei	40.5	Smith_etal_2003	.
Odontoceti	Platanistidae	Pontoporia blainvillei	34	Jefferson_Leatherwood_Webber_1993	.
Odontoceti	Ziphiidae	Berardius arnuxii	7000	Smith_etal_2003	.
Odontoceti	Ziphiidae	Berardius bairdii	11380	Smith_etal_2003	.
Odontoceti	Ziphiidae	Berardius bairdii	12000	Jefferson_Leatherwood_Webber_1993	.
Odontoceti	Ziphiidae	Hyperoodon ampullatus	5800	Smith_etal_2003	.
Odontoceti	Ziphiidae	Hyperoodon planifrons	3000	Smith_etal_2003	.
Odontoceti	Ziphiidae	Indopacetus pacificus	2200	Smith_etal_2003	.
Odontoceti	Ziphiidae	Mesoplodon bidens	3400	Smith_etal_2003	.
Odontoceti	Ziphiidae	Mesoplodon bowdoini	2600	Smith_etal_2003	.
Odontoceti	Ziphiidae	Mesoplodon carlhubbsi	1400	Jefferson_Leatherwood_Webber_1993	.
Odontoceti	Ziphiidae	Mesoplodon carlhubbsi	3400	Smith_etal_2003	.

Odontoceti	Ziphiidae	Mesoplodon carlhubbsi	500	Mean_Walker_Houck_1982	.
Odontoceti	Ziphiidae	Mesoplodon densirostris	2300	Smith_etal_2003	.
Odontoceti	Ziphiidae	Mesoplodon densirostris	1033	Jefferson_Leatherwood_Webber_1993	.
Odontoceti	Ziphiidae	Mesoplodon europaeus	5600	Smith_etal_2003	.
Odontoceti	Ziphiidae	Mesoplodon europaeus	1200	Jefferson_Leatherwood_Webber_1993	.
Odontoceti	Ziphiidae	Mesoplodon ginkgodens	1500	Smith_etal_2003	.
Odontoceti	Ziphiidae	Mesoplodon grayi	2900	Smith_etal_2003	.
Odontoceti	Ziphiidae	Mesoplodon grayi	1100	Jefferson_Leatherwood_Webber_1993	.
Odontoceti	Ziphiidae	Mesoplodon hectori	1000	Smith_etal_2003	.
Odontoceti	Ziphiidae	Mesoplodon layardii	1500	Smith_etal_2003	.
Odontoceti	Ziphiidae	Mesoplodon mirus	2100	Smith_etal_2003	.
Odontoceti	Ziphiidae	Mesoplodon mirus	1400	Jefferson_Leatherwood_Webber_1993	.
Odontoceti	Ziphiidae	Mesoplodon mirus	1400	Culik_2004	.
Odontoceti	Ziphiidae	Mesoplodon stejnegeri	4800	Smith_etal_2003	.
Odontoceti	Ziphiidae	Tasmacetus shepherdi	2500	Smith_etal_2003	.
Odontoceti	Ziphiidae	Ziphius cavirostris	4775	Smith_etal_2003	.
Odontoceti	Ziphiidae	Ziphius cavirostris	3000	Jefferson_Leatherwood_Webber_1993	.
